# Metagenome-Assembled Genome Sequences Obtained from a Reactivated Kombucha Microbial Community Exposed to a Mars-Like Environment outside the International Space Station

**DOI:** 10.1128/MRA.00549-21

**Published:** 2021-09-09

**Authors:** Imchang Lee, Debmalya Barh, Olga Podolich, Bertram Brenig, Sandeep Tiwari, Vasco Azevedo, Aristóteles Góes-Neto, Oleg Reva, Natalia Kozyrovska, Jean-Pierre de Vera, Bong-Soo Kim

**Affiliations:** a Department of Life Science, Multidisciplinary Genome Institute, Hallym University, Chuncheon, Republic of Korea; b Institute for Liver and Digestive Diseases, Hallym University, Chuncheon, Republic of Korea; c Institute of Integrative Omics and Applied Biotechnology (IIOAB), Nonakuri, Purba Medinipur, West Bengal, India; d Institute of Molecular Biology and Genetics of NASU, Kyiv, Ukraine; e Institute of Veterinary Medicine, University of Göttingen, Göttingen, Germany; f Laboratory of Cellular and Molecular Genetics, Department of General Biology, Institute of Biological Sciences, Federal University of Minas Gerais, Belo Horizonte, Brazil; g Molecular and Computational Biology of Fungi Laboratory, Department of Microbiology, Institute of Biological Sciences, Federal University of Minas Gerais, Belo Horizonte, Brazil; h Centre for Bioinformatics and Computational Biology, Department of Biochemistry, Genetics and Microbiology, University of Pretoria, Pretoria, South Africa; i Microgravity User Support Center (MUSC), German Aerospace Center (DLR), Cologne, Germany; University of Arizona

## Abstract

Kombucha is a traditional tea fermented by symbiotic microbiota, and it has been known as a functional fermented product. Here, we report four microbial metagenome-assembled genome sequences (MAGs) reconstructed from the microbiomes in kombucha exposed to a Mars-like environment outside the International Space Station.

## ANNOUNCEMENT

Kombucha is a traditional sugared tea that is fermented using a symbiotic community of acetic acid bacteria and osmophilic acid-tolerant yeasts. Kombucha microbial community (KMC) samples, composed of bacterial and eukaryotic microorganisms, were used as a part of the BIOMEX (BIOlogy and Mars EXperiment) project to understand the influence of exposure to spaceflight and Mars-like conditions on microbiota (1, 2).

Dried KMC pellicles of ecotype IMBG-1 (from the collection of the Institute of Molecular Biology and Genetics of NASU (IMBG), Kyiv, Ukraine) were exposed to Mars-like conditions simulated on low Earth orbit in the three-layer sample carrier mounted on the EXPOSE-R2 facility outside the International Space Station (ISS) (1). During a 2.5-year exposure at the ISS (18 months outside the station and 7 months inside), samples located on the unprotected top level received UV rays (>200 nm), cosmic ionizing radiation, and other fluencies (2). The middle and bottom levels were protected from UV radiation; however, they were maintained in the Mars-like atmosphere and pressure. During the exposure period, all the KMC samples were kept dried with temperature fluctuations. Analogically prepared KMC samples were maintained under laboratory conditions as references.

The KMC samples exposed to Mars-like conditions and laboratory references were reactivated as previously described (1). The growth rates, cellulose-based pellicle yields, and enzyme activities in the KMC samples were different from those in the ground reference samples after reactivation (3, 4). The KMC specimens that had been exposed to Mars-like UV radiation showed major functional alterations compared to the UV-protected samples. However, the genes sequenced from the dominant strains in the KMCs remained unaltered, meaning that the kombucha samples were ecologically resilient. Therefore, we analyzed the metagenome-assembled genome sequences (MAGs) of the dominant species to understand the effect of space/Mars-like conditions on the microecosystem of the KMCs.

For the shotgun metagenomic analyses, we used the returned reactivated KMCs (exposed on the three-level-sample carrier), as well as the initial KMC ecotype, which had been saved for sample preparation in the space experiment, and the laboratory kept the desiccated KMC samples used as references (1, 5). For the metagenomic DNA extraction, the KMC samples were cultured in 50 ml black tea-sugar medium until the stage of biofilm formation (5 days), as described in a previous study (5). The biofilm pellicle was removed, and planktonic cells were precipitated using a centrifuge.

Metagenomic DNA was extracted from the planktonic cells of the KMC samples using the innuSPEED bacteria/fungi DNA isolation kit (Analytik Jena AG, Germany). A sequencing library was prepared using a NEBNext Fast DNA fragmentation and library preparation kit (New England BioLabs, Ipswich, MA, USA). Paired-end sequencing (2 × 150 bp) was performed using a HiSeq 2500 instrument (Illumina, USA).

The raw sequences were filtered for quality control using SolexaQA (6), and the low-quality sequences and artificially duplicated reads (ARDs) were removed using Illumina-Utils (7) and duplicate read inferred sequencing error estimation (DRISEE) (8), respectively. A total of 497,559,912 reads (74,633,986,800 bp) were assembled using MEGAHIT v1.2.9 (9), resulting in 7,303 metagenomic contigs with a length of 65,241,169 bp. After removal of the short reads (<1,000 bp), a total of 1,435 contigs were used for the following binning process. The MAGs were reconstructed using the Anvi-refine program in Anvi’o v6.2 (10). The metagenomic contigs were binned based on the single-copy core gene (SCG) set of Kaiju v1.6.2 (11) in the Anvi’o environment. The completeness and contamination of the MAGs were determined using CheckM v1.1.3 (12). Only the MAGs with a completeness of more than 90% and a contamination rate of less 5% were considered for further analysis. Four bacterial MAGs were obtained after a quality check, and the taxonomic classification was detailed by comparing the average nucleotide identity (ANI) with closely related strains in the NCBI Assembly database using the OrthoANI tool (13). Closely related strains were selected based on the binning results. Default parameters were used for all software unless otherwise specified. Three MAGs were classified as Komagataeibacter, and one MAG was classified as Pseudomonas. The MAG information is summarized in Table 1. The genome sizes of the three Komagataeibacter MAGs ranged from 3,326,596 to 4,214,122 bp, and the GC contents ranged from 60.14% to 63.35%. The genome size of the Pseudomonas MAG was 6,200,971 bp, and the GC content was 60.57%. Additional genome characterization and functional annotation were automatically performed using the Prokaryotic Genome Annotation Pipeline (PGAP) (14). These MAGs will provide insights into the effect of Mars-like conditions on the microbes in kombucha.

**TABLE 1:** General features of metagenome-assembled genomes from kombucha microbial community

MAG	Taxonomic classification	Genome size (bp)	No. of contigs	No. of genes	*N*_50_ (bp)	GC content (%)	CMP^*a*^ (%)	CNT^*a*^ (%)	Mean coverage (×)	BioSample accession no.	GenBank accession no.
kmcMAG001	Komagataeibacter hansenii	4,214,122	108	3,779	79,588	60.14	94.65	4.98	138.26	SAMN16843767	JAEORC000000000
kmcMAG002	Komagataeibacter rhaeticus	3,326,596	90	3,040	62,857	63.35	99.11	1.69	26.01	SAMN16843789	JAEORD000000000
kmcMAG003	Komagataeibacter oboediens	3,370,429	63	3,102	88,723	62.04	98.77	1.49	47.65	SAMN16843790	JAEORE000000000
kmcMAG004	Pseudomonas sp.	6,200,971	143	5,716	66,800	60.57	97.69	2.41	2.31	SAMN16931596	JAEORF000000000

a The completeness (CMP) and contamination (CNT) were determined using CheckM v1.1.3.

### Data availability.

The raw sequence data were deposited at the National Center for Biotechnology Information (NCBI) database under the project numbers PRJNA636820, PRJNA636837, PRJNA636891, PRJNA637016, and PRJNA637018. The MAGs are available at GenBank under the BioSample accession numbers SAMN16843767, SAMN16843789, SAMN16843790, and SAMN16931596 and under the accession numbers JAEORC000000000, JAEORD000000000, JAEORE000000000, and JAEORF000000000, respectively.
